# Vaginismus

**Published:** 1887-02

**Authors:** Geo. E. Fell

**Affiliations:** Physician to Sisters of Charity Hospital, Professor of Physiology and Microscopy, in Medical Department of Niagara University, Treasurer and Custodian American Society Microscopists, etc.


THE BUFFALO MEDICAL AND SURGICAL JOURNAL

Vol. XXVI. FEBRUARY, 1887. No. 7

### Original Communications.

### *VAGINISMUS.* *

BY GEO. E. FELL, M. D., F. R. M. S.,

Physician to Sisters of Charity Hospital, Professor of Physiology and Microscopy, in Medical Department of Niagara University, Treasurer and Custodian American Society Microscopists, etc.

The condition of the generative organs of the female, which I have the honor of presenting for your consideration this evening, is one of extreme interest as affecting the welfare of not only the virgin, as in the case I will relate, coming under my own experience, but to both parties to the married state, when the condition prevails, as it very often does in the married woman.

Furthermore, I believe a paper upon this subject has not yet been presented to our society.

To a no less high authority than Dr. Marion Simms do we owe the term *Vaginismus*, which, as accepted, implies an affection of the nerves of the vagina in the region of the hymen, marked by contraction of the muscles in and about the vaginal walls, which interferes, to a certain extent, with the function of these parts, and often seriously modifies the health of the patient.

* Read before the Buffalo Obstetrical Club, Oct, 27, 1886.

There is some difference of opinion regarding the pathology of vaginismus; while it is conceded to be a neurosis, all are not agreed as to the muscular factors which produce the stricture. Lusk, in speaking of the structure of the vagina, says, "The muscular fibres, which are of the involuntary variety, run in both a longitudinal and transverse direction, and are so interwoven together that a dissection into distinct strata is impossible." Further, "that the muscular layers gradually increase in thick ness as they approach the vaginal orifice, and, according to Luschka, a circular bundle of voluntary fibres, the sphincter vaginæ, surrounds the lower extremity of the vagina and urethra." In this statement, it is noted that the muscular fibres of the vagina appear to be the constrictor factors. Dr. Thomas says, "It is generally accepted as a fact that the bulbo-cavernosus muscle, which passes over the clitoris and forms a figure of 8 with the sphyncter ani, is the constrictor vaginæ." Dr. Savage denies this, and claims that the pubo-coccygeus muscle is the main constrictor in vaginismus. This muscle is situated within the pelvis just above the point at which the vaginal walls branch off to seek their osseous attachment. It arises from the inner surface of the pubic bone. Its median fibres descend by the side of the urethra and vagina, some of them turning in between the vagina and rectum to meet similar fibres from the opposite side in the perineal body; another more outward series, turning in beneath the rectum, intermix with fibres from the other side; while the remaining, still more outward, are inserted into the side of the coccyx (Thomas, p. 142). Gray, in speaking of the sphincter vaginæ, says it surrounds the orifice of the vagina—is attached, posteriorly, to the central tendon of perineum, where it blends with the sphincter ani, and that its fibres pass forward on each side of the vagina, to be inserted into the corpora cavernosa of the clitoris. This last muscle corresponds in its location and general description to the bulbo-cavernosus previously described, and, while it may be looked upon as the chief factor in constricting the vagina, it is altogether probable that the deeper muscles corresponding to the pubo-coccygeus are also brought into play in this affection.

The causes which are said to produce this condition are almost as numerous as the diseases which affect the female genitalia.

The hysterical condition is said to be a very common factor, and yet, according to authorities of value, the treatment adopted frequently cures, or, at least, wonderfully modifies hysteria.

HYSTERICAL VAGINISMUS.

Thomas, (Diseases of Women, p. 143,) gives the recognized causes of this disease as follows:

The hysterical diathesis.

Excoriations or fissures at the vulva.

Irritable caruncle of the meatus.

Chronic endometritis or vaginitis.

Pustular or vesicular eruptions on the vulva.

Hyperæsthesia of the remains of the hymen.

An abnormally rigid perineum.

Disproportionately large size of male organ.

Others ascribe it to fissure of the anus.

The views of Prof. Scanzoni, who had a large experience with this disease, are instructive.

Of thirty-four cases, he found that twenty-five had various functional and organic difficulties, which in twenty cases had come on after marriage.

In eleven, there was congestive dysmenorrhoea.

In one, amenorrhœa had existed for three years.

In thirteen, there was chronic metritis.

Four had either retro or ante version.

In one, there was perimetritis.

In seventeen, chronic uterine catarrh.

In fourteen, vaginal catarrh.

In one, anteflexion.

In two, retroflexion.

Nine had urinal difficulties.

One had inflammation of the right Bartholian's gland.

In fourteen, there was symptoms of anæmia, and in seventeen there was hysteria.

According to Frankenhauser, (Hart and Barbour, p. 74,) "the terminations of the nerves in the muscular layers of the uterus is in the nuclei of the unstriped muscle;" this may be the condition in the vagina. He states also "that numerous end bulbs have been found in the clitoris and vagina." It is not unreasonable to suppose that any abnormality producing a hyperæsthetic condition of these nerve terminals, would, through reflex action, cause vaginismus.

*Diagnosis.*—It would seem from the very nature of the disease that there would be no difficulty in diagnosis, if opportunity is offered the physician to make an examination.

In the young married woman with such a condition, the husband is quite likely to soon have the case referred to the physician, but in the virgin it is not so easy to bring this about, and many steps are taken before an examination is demanded. A proper digital examination should, in my opinion, make clear the diagnosis.

*Prognosis.*—Dr. Simms* says, "I can confidently assert that I know of no disease capable of producing so much unhappiness to both parties to the marriage contract, and I am happy to state that I know of no serious trouble that can be so easily, so safely and so certainly cured."

*Treatment.*—One writer says the treatment after marriage is *psyhcical*, discontinue coitus and with increased mutual confidence everything will be right later on. He also speaks of slightly stretching with dilators. (Fritch, "Diseases of Women," pp. 101 and 102).

Tait ("Diseases of Women," p. 46,) says, "The great majority of these cases are cured by the use of simple cerate and the restriction of intercourse within moderation. Further, that of the large number of cases sent to him as vaginismus, for the purpose of having the operation of division of the sphincter muscle performed, he had not operated upon for the reason that he had been able to find a more tangible cause for the patient's

* Simms' Uterine Surgery, p. 326.

distress than the **hypothetical* contraction of the sphincter muscle." As to the hypothetical contraction of the sphincter muscle, I should be constrained to think that the writer had never experienced the sensation of a sphincter vagina contracting on his finger, or he would have used no such expression.

Hart and Barbour (Manual of Gynecology,) recommend the removal of any carunculae which may exist, the division of the base or touching of irritable fissures with the actual cautery; the use of iodoform ointment, and then, after the cause is removed the ostium vaginæ must be dilated. If this does not accomplish the object, we must have recourse to Simms' operation.

"Dr. Tait (Diseases of Women,) recommends the chloroform anesthesia of the patient and the introduction of the two thumbs into the vagina, back to back, and their forcible separation, so as to keep the vagina distended for a few minutes." On the same principle Dr. W. P. Hood, under chloroform narcosis, dilated the vagina by the expansion of a bivalve speculum, and allowed the instrument to remain there five minutes.

The operation of Dr. Simms is performed as follows: The patient under anæsthetics, on her back upon a table, the remains of the hymen are entirely excised with a pair of curved scissors. Hemorrhage is prevented with cold applications (ice-water,) or persulphate of iron.

The index and middle finger of the left hand are then passed into the vagina, putting the fourchette on the stretch. By means of a scalpel, a deep incision is then made of the right of the mesial line, terminating at the raphæ of the perineum. A similar incision is then made on the opposite side, the two being united at the raphæ, and extended to the perineal integument and through its upper border. Each of these incisions will extend from about half an inch above the upper border of the sphincter to the perineal raphæ, thus passing across the muscle, and measuring nearly two inches. The vaginal dilator is worn for a few hours in the morning and evening, according to the

* Italics mine. G. E. F.

tolerance for which it is manifested. Another operation endorsed by a high authority, but not generally approved by the writers upon this subject, is one proposed by a Dr. Burns, and consists in section of the pudic nerve. This is mentioned merely to condemn it.

Dr. Thomas (Diseases of Women, p. 149), recommends in the light of our knowledge at present on the subject:

1st—The removal of disease of the genitalia, if such be found; insists upon the patient living apart from her husband; recommends copious injections of warm water twice daily; the use of local suppositories, tonics, change of air, sea bathing, and the introduction of the glass plug in the vagina for several hours each day.

2d—Should this not suffice, anæsthetize the patient, and by means of a tri-valve, or quadri-valve speculum (the latter I believe to be best), distend the ostium vaginæ thoroughly; follow with the use of the vaginal plug and soothing applications.

3d—Should this also fail, with the patient under anæsthetics, remove the hymen with scissors, incise the perineum as it is torn in parturition, and use the vaginal plug for a week.

This treatment is that which appeals most strongly to our reason, and will undoubtedly almost always prove effectual.

The majority of the authorities oppose the use of the knife, and, owing to the dangers incurred in incisions about these most vascular parts, we cannot but agree with them. Barnes states that he has cured many cases by the methods adopted by Scanzoni—" the allaying of inflammation that may exist, and the gradual dilation by glass plugs worn for short intervals at a time—" but that he has met with cases where the knife or scissors gave *not only* the *quickest* and most *efficient relief*, but also it was accomplished at the least cost of pain and other distress.

REPORT OF CASES.

Hewitt* gives an instance of a case in a married lady who had had two children; for some months there had been extreme

* Hewitt, "Diseases of Women," third edition, 1880.

sensibility of the ostium vaginæ, preventing intercourse. He found that the sensibility was limited to a spot near the posterior commisure, over an area of less than one quarter of an inch. The mucous membrane over this part was pared away, the lips of the wound sutured with fine silver wire, with an abatement of the trouble.

I might cite many cases reported by gynecologists of extensive practice; they would be of no special value, as after a consideration of the causes, and general methods of treatment, it will be seen that the judgment of the practitioner is a most important factor.

In conclusion, I will report an interesting case which came before me.

Miss B., aged fifteen, was found to be in a hysterical condition at times quite violent, but generally controllable. The paroxysms had increased within the last few months, and taken such peculiar manifestations, that she had become a great trial to her parents and friends. The attacks were ushered in by a sudden outburst of anger, a desire to bite her attendants, this would pass away, the patient become laughable, and when opportunity appeared to offer, the biting would be renewed. When overpowered by the attendant, she would frequently quietly give up with that peculiar glaring, laughing, meaningless smile so frequently seen in the hysterical woman. Tonic spasms would ensue at frequent intervals with the ordinary characteristics; in a few minutes they would pass away, leaving the patient physically prostrated for the time being. This continued for some time. Three physicians had seen the case, two recommending chloral and the bromides; the third placed the patient under the influence of chloroform kept up the anæsthesia for a short time, without any apparent definite purpose in view, allowed her to pass from under its effect, and gave up the case.

As I viewed this case, my mind ran upon the fact that one of the most frequent causes of perturbed nervous action in the young female was undoubtedly abnormalities associated with the menstrual function; in this case, menstruation was not yet established, I thought—possibly delayed and a condition of dysmenorrhœa existing. To relieve the patient, vaginal suppositories of opium and belladonna were ordered with most satisfactory results. It was noted, however, that they could with difficulty be passed into the vagina by the patient.

Concluding that a digital or specular examination was requisite, I so informed the parents of the young lady, and on the following day made it with satisfactory results. Excepting entirely the natural modest objections on the part of the patient to an examination of this nature, the paroxysms produced by attempting to pass the finger into the vagina were of the most violent kind; indications of acute pain, evidenced by terror on the part of the patient, were noted whenever attempts were made to pass the finger through the sphincter.

The contraction of the vagina began about one-half inch from the ostium, and, as near as could be determined, was fully an inch in extent, indicating that the deeper muscles were involved. The middle finger could not be made to enter; the little finger was used with better results, passing, after some effort, to the vaginal cul de sac. The suppositories were kept up for a day, until they lost their efficacy, the nervous condition of the patient having been greatly aggravated by the examinations referred to. A series of spasms set in the third day of a most violent nature, one rapidly following the other, until the patient became so much exhausted that immediate operative interference was decided upon. Assisted by Drs. Daniels and Smith, the patent was placed under the following anæsthetic :

| | | |
|---|---|---|
| ℞ | Chloroformi, - - - | fld. ℥ iii. |
| | Ether (Squibbs'), - - | fld. ℥ iss. |
| | Alcohol, - - - - | fld. ʒ ii. |

This combination has given, in a number of cases, such great satisfaction that I will ask for no better. and deem it worthy of mention in this connection. After complete anæsthesia had been produced, a four-bladed anal speculum was introduced, closed, to the fundus vaginæ. The instrument was gradually dilated until the blades were separated from one and one-quarter to one and three-quarter inches, the dilatation held at this extreme extent for a few minutes, and the blades of the instrument closed; this was repeated, a vaginal suppository (opium and belladonna) introduced, and a glass plug an inch in diameter placed in the vaginæ, and retained with a "T" bandage. While the speculum was in the vigina, the os uteri was dilated, the cavity of the uterus examined, but no abnormalities appeared to exist.

The only change in proceedure from that mentioned above that I should suggest, would be to dilate quite as fully and retain the vagina in the dilated state for a greater length of time—say ten to fifteen minutes—then use the glass plugs.

The results in this case were sufficiently satisfactory to attest the value and necessity of the method of treatment. The hysterical condition was immediately abated, and while another dilatation was recommended, to insure a permanent improvement, the results have been so satisfactory that the method of treatment is demonstrated to be the only one which will prove of any *lasting* benefit to the patient.

Furthermore the value of a single dilatation is evidenced in the greater susceptibility of the patient, thereafter, to the influence of treatment by drugs. Since the operation was performed menstruation has set in and the condition of the patient is markedly improved.

